# Correction: Becoming the temporary surgeon: A grounded theory examination of anaesthetists performing emergency front of neck access in inter-disciplinary simulation-based training

**DOI:** 10.1371/journal.pone.0251285

**Published:** 2021-04-29

**Authors:** The PLOS ONE Staff

## Notice of republication

This article was republished on April 22, 2021, to correct an error in the Participants subsection of the Methods introduced in the typesetting process. The publisher apologizes for the error. Please download this article again to view the correct version. The originally published, uncorrected article and the republished, corrected article are provided here for reference.

## Supporting information

S1 File Originally published, uncorrected article. (PDF)

S2 File Republished, corrected article. (PDF)
