# Supplementary material for: Becoming the temporary surgeon: A grounded theory examination of anaesthetists performing emergency front of neck access in inter-disciplinary simulation-based training
Source: PLoS One. 2021 Mar 23;16(3):e0249070. doi: 10.1371/journal.pone.0249070 (PMC7987190; doi:10.1371/journal.pone.0249070)
Supplement: Attachment — Submitted filename: Response to Reviewers - Jan2021.docx [file pone.0249070.s001.docx]

| **Editor’s Comments** | | |
| --- | --- | --- |
| 1 | **Thank you for submitting your manuscript to PLOS ONE. After careful consideration, we feel that it has merit but does not fully meet PLOS ONE’s publication criteria as it currently stands. Therefore, we invite you to submit a revised version of the manuscript that addresses the points raised during the review process.**    **• Please address the reviewers' comments**  **• The manuscript is too lengthy. It would be easier to be followed if it has been abstracted**  **Thank you very much for your interest in the Plos One. The two expert reviewers have raised interesting suggestions to improve the quality of the manuscript. Can you please kindly address all comments in the next version of the manuscript. I would highlight the followings**  **1. The generalizability and logistics for including FONA in the curriculum of training in anaesthesia.**  **2. The need to reduce the length of the manuscript.** | We would like to thank you and the reviewers for your positive appraisals of our manuscript and helpful suggestions for ways to revise the manuscript. We have worked hard to address all the points raised in the reviews.  We have worked through all the reviewers’ comments and have revised accordingly.  We have significantly shortened the length of the manuscript by approximately 2,000 words from almost 9,000 words (introduction to conclusion, inclusive; exclusive of title page, abstract, and references) to 6,887 (introduction to conclusion, inclusive; exclusive of title page, abstract, and references).  We would like to thankyou for your positive commentary on our paper. We have taken the time to address all comments made by the reviewers and editors.  Thankyou for this comment, we realise this was also raised by Reviewer 1. We have clarified in the intervention development section that 6-month follow-up was for the purposes of the research evaluation only. We have also added to the implications section how this could be scaled-up.  We have significantly shortened the length of the manuscript from 8,755 words (introduction to conclusion, inclusive; exclusive of title page, abstract, and references) to 6,737 (introduction to conclusion, inclusive; exclusive of title page, abstract, and references). |
| **Journal Requirements** | | |
| 2 | **Please ensure that your manuscript meets PLOS ONE's style requirements, including those for file naming.** | We have now done this. |
| 3 | **It is unclear why 'No - some restrictions will apply' ; please clarify the nature of these restrictions, ie. If due to ethical or legal reasons.**  **PLOS only allows data to be available upon request if there are legal or ethical restrictions on sharing data publicly.** | We have now rectified this on the on-line submission system. |
| 4 | a) **If there are ethical or legal restrictions on sharing a de-identified data set, please explain them in detail (e.g., data contain potentially identifying or sensitive patient information) and who has imposed them (e.g., an ethics committee). Please also provide contact information for a data access committee, ethics committee, or other institutional body to which data requests may be sent.** b) **If there are no restrictions, please upload the minimal anonymized data set necessary to replicate your study findings as either Supporting Information files or to a stable, public repository and provide us with the relevant URLs, DOIs, or accession numbers.**   **We will update your Data Availability statement on your behalf to reflect the information you provide.** | We have updated the online submission platform to reflect the following:  *Data cannot be shared publicly because of the identifiable nature of the interview transcripts. Data are available from the Health Research Institute Institutional Data Access at Edge Hill University (contact via Sergio A. Silverio: Sergio.Silverio@kcl.ac.uk and Jeremy Brown: Brownjm@edgehill.ac.uk) for researchers who meet the criteria for access to confidential data.* |
| 5 | **Thank you for your ethics statement:**  **'Ethical approvals were sought from and granted by Health Research Authority with IRAS project ID: 226720 and REC reference: 18/HRA/0122, with both University and Hospital permissions, subsequently granted.'**  **a. Please amend your current ethics statement to include the full name of the ethics committee/institutional review board(s) that approved your specific study.**  **b. Once you have amended this/these statement(s) in the Methods section of the manuscript, please add the same text to the “Ethics Statement” field of the submission form (via “Edit Submission”).** | We have now addressed this issue.  We have now added the full names of each of the boards/committees.  We have uploaded this to the Ethics Statement section of the submission platform. |
| 6 | **Please provide additional details regarding participant consent.**  **In the ethics statement in the Methods and online submission information, please ensure that you have specified (i) whether consent was informed and (ii) what type you obtained (for instance, written or verbal, and if verbal, how it was documented and witnessed). If your study included minors, state whether you obtained consent from parents or guardians. If the need for consent was waived by the ethics committee, please include this information.**  **If you are reporting a retrospective study of medical records or archived samples, please ensure that you have discussed whether all data were fully anonymized before you accessed them and/or whether the IRB or ethics committee waived the requirement for informed consent. If patients provided informed written consent to have data from their medical records used in research, please include this information.** | This has now been written into the manuscript and uploaded on the on-line submission platform. |
| 7 | **Thank you for stating the following in the Competing Interests section:**  **'Sergio A. Silverio (King’s College London) is supported by the National Institute for Health Research Applied Research Collaboration South London [NIHR ARC South London] at King’s College Hospital NHS Foundation Trust. The views expressed are those of the authors and not necessarily those of the NIHR or the Department of Health and Social Care. '**    **a. Please confirm that this does not alter your adherence to all PLOS ONE policies on sharing data and materials, by including the following statement: "****This does not alter our adherence to PLOS ONE policies on sharing data and materials.” If there are restrictions on sharing of data and/or materials, please state these.**  **Please note that we cannot proceed with consideration of your article until this information has been declared.**    **b. Please include your updated Competing Interests statement in your cover letter; we will change the online submission form on your behalf.**  **Please know it is PLOS ONE policy for corresponding authors to declare, on behalf of all authors, all potential competing interests for the purposes of transparency. PLOS defines a competing interest as anything that interferes with, or could reasonably be perceived as interfering with, the full and objective presentation, peer review, editorial decision-making, or publication of research or non-research articles submitted to one of the journals. Competing interests can be financial or non-financial, professional, or personal. Competing interests can arise in relationship to an organization or another person.** | Thankyou for bringing this to our attention, our amendments are detailed below.  This statement has now been added to the declaration.  We have included all our declarations in the cover letter. |
| **Reviewer 1** | | |
| 8 | **Reviewer #1: Thanks for this interesting study and study approach;**  **however, there are some concerns.** | We would like to thank the reviewer for their positive commentary on our manuscript and for their helpful suggestions. We have now undertaken these changes and believe we have improved the manuscript accordingly. |
| 9 | **I do support the idea to use structured interviews and identify frames of action the study participants. However the manuscript has an incredible length; as well documented, adults (and even kids with different devices) can use scales to grade certain expressions describing feelings / ideas etc. this might help to grade the progress in the training. The bites from the interviews are interesting to read, but too long ; you should put those in a supplemental file** | We thank the reviewer for their suggestion. We have now significantly reduced the amount of quotations, with many being added into the table. |
| 10 | **I understand that this longitudinal study in this setting cannot be designed to train hundreds of residents. Is you approach applicable for larger groups?** | Thankyou for this comment. We have clarified in the intervention development section that 6-month follow-up was for the purposes of the research evaluation only. We have also added to the implications section how this could be scaled-up. |
| 11 | **Please straighten the manuscript; it is way too long; this accounts for all parts of the document.** | We thank the reviewer for their comment and have made considerable reductions to every part of the text in-line with your suggestion. |
| **Reviewer 2** | | |
| 12 | **Congratulations for the very interesting study.** | Thankyou very much. We are very grateful for such positive appraisal, and have incorporated changes in-line with your comments. |
| 13 | **I have just one suggestion, although it is very probably not even fairly related to the context.**  **First of all, the addition of FONA into the curriculum of anaesthesia should be set a precedent in other countries. During the process I think one of the most important steps is medico-legal and more importantly "self-ethical" issues. (To explain my concern: If an anaesthesist has to apply a FONA, she/he should have no concerns about the possible question "if I do something wrong, will I be sued?";**  **and in the second , more important step, the more important question: "if I do something wrong, will I ask myself that it would be better to let the surgeon do it?"....**  **I think these questions deserve also to be discussed.** | We would like to thank the reviewer for their suggestions and for accepting these may be outside the scope of the paper. We have however made reference to each issue in the paper.  We realise this will differ from one place to the other across the world. We can only answer from a UK point of view with sympathies for other countries. Fear of litigation may be another factor in the evaluation of this training, more so than the UK and we have now alluded to this in the limitations section of our manuscript.  This study was not about who was best to perform FONA. We recognise there are many incidences in UK where variety of doctors perform FONA depending on skills and expertise in the team. This training was about how Anaesthetist feel and were trained to do it. Whoever does it rightly/wrongly presumes themselves as the best person to do it. This study should make Anaesthetist better equipped to do it – and that was the scope of this study. No doubt that this is a fundamental question. The bottom line with FONA is that it does not provide the luxury to plan – so in that instance it is the most qualified/trained person in the room to do it and with this training could be the Anaesthetist. Anaesthetist are trained to know the hierarchy of who should perform FONA first. We have made reference to this in our conclusion |
